# Transient Headache and Neurological Deficits with cerebrospinal fluid Lymphocytosis (HaNDL) syndrome in children: case report and narrative review

**DOI:** 10.1186/s13052-025-02139-9

**Published:** 2025-11-19

**Authors:** Giulia Abrate, Roberta Rossi, Giulia Grasso, Barbara Lauria, Cristina Vassia, Emanuele Castagno, Claudia Bondone, Antonia Versace

**Affiliations:** 1 Department of Public Health and Pediatrics, Postgraduate School of Pediatrics, University of Turin, Piazza Polonia 94, Turin, 10126 Italy; 2 Department of Pediatric Emergency - Pediatric Headache Centre, Regina Margherita Children’s Hospital, Piazza Polonia 94, Turin, 10126 Italy

**Keywords:** HaNDL syndrome, Cerebrospinal fluid lymphocytosis, Children, Headache, ICHD-3

## Abstract

**Background:**

The transient Headache and Neurological Deficit with cerebrospinal fluid Lymphocytosis (HaNDL) Syndrome is a rare form of primary headache, with few cases reported in children.

**Case presentation and review:**

We report the case of a 15-year-old female with HaNDL syndrome showing paresthesia to the right side of her face and upper limbs, asthenia, dysarthria and aphasia, followed by left periorbital pulsating headache with moderate nausea, lasting about four hours. Forty-four cases of pediatric HaNDL syndrome are reported in literature (ours included), but only 25 fulfilled all diagnostic criteria according to ICHD-3. Overall, 59.1% were females. Sensory symptoms affected 71.4% of patients, followed by impaired speech (69.0%) and motor symptoms (52.4%). At CSF analysis, the mean value of white blood cells was 201.1/µl; proteinorrhachia was reported in 31 patients (70.5%). When asked, neuroimaging was negative. Symptomatic treatment was reported only in 14 patients (31.8%).

**Conclusions:**

HaNDL is a rare self-limiting syndrome affecting both adults and children. The etiology is unknown, but autoimmune mechanisms have been proposed. HaNDL is a diagnosis of exclusion: differential diagnoses include stroke, tumors, epilepsia, neuro-infective disorders, autoimmune encephalitis, vasculitis, hemiplegic migraine and migraine with aura. Usually, HaNDL episodes last less than 3 months; therapy is symptomatic. The diagnostic work out includes CSF analysis, neuroimaging and EEG. The treatment is symptomatic, and the course is self-limiting, usually resolving within 3 months.

**Supplementary Information:**

The online version contains supplementary material available at 10.1186/s13052-025-02139-9.

## Background

The transient Headache and Neurological Deficit with cerebrospinal fluid Lymphocytosis (HaNDL) Syndrome is a rare form of primary headache. Only a few cases have been reported in children so far. We describe the case of a 15-year-old female affected by HaNDL, followed by a review of the literature of pediatric cases.

## Case presentation

A previously healthy 15-year-old female was admitted to our Emergency Department (ED) because of paresthesia to the right side of her face and upper limbs, asthenia, dysarthria and aphasia, lasting about two hours, followed by left periorbital pulsating headache with moderate nausea, lasting about four hours. In recent medical history, gastroenteritis was reported few weeks before; past medical history was unremarkable, except for mild occasional headache during febrile episodes. Her parents reported occasional headache and maternal grandmother suffered from migraine.

At the entry, the girl was in good general condition, afebrile, with no acute neuropathological signs in progress, asymptomatic for headache, and with negative neurological examination. Head computed tomography (CT), CT angiography and blood tests including complete blood count, C-reactive protein, liver and kidney function, and coagulative profile proved negative. CSF analysis showed lymphocytosis (leukocytes 127/µl, 100% lymphocytes), slight proteinorrhachia (total proteins: 622 mg/l; albumin: 403 mg/l), no glycorrachia, and negative Polymerase Chain Reaction for viruses and bacteria. CSF opening pressure was not measured. On suspicion of encephalitis, she was firstly put on intravenous ampicillin, acyclovir and dexamethasone. Antibiotic therapy was then stopped after confirming negative blood count and C-reactive protein 24 h later. Further investigations included electroencephalogram (EEG) and head magnetic resonance imaging (MRI) with MRI angiography, all proving negative; antiviral and steroid therapy were then suspended 48 h after the onset. Also, thrombophilia profile and autoantibodies for dysimmune encephalitis (anti-NMDA and anti-GAD) on cerebrospinal fluid were negative.

**Fig. 1:**
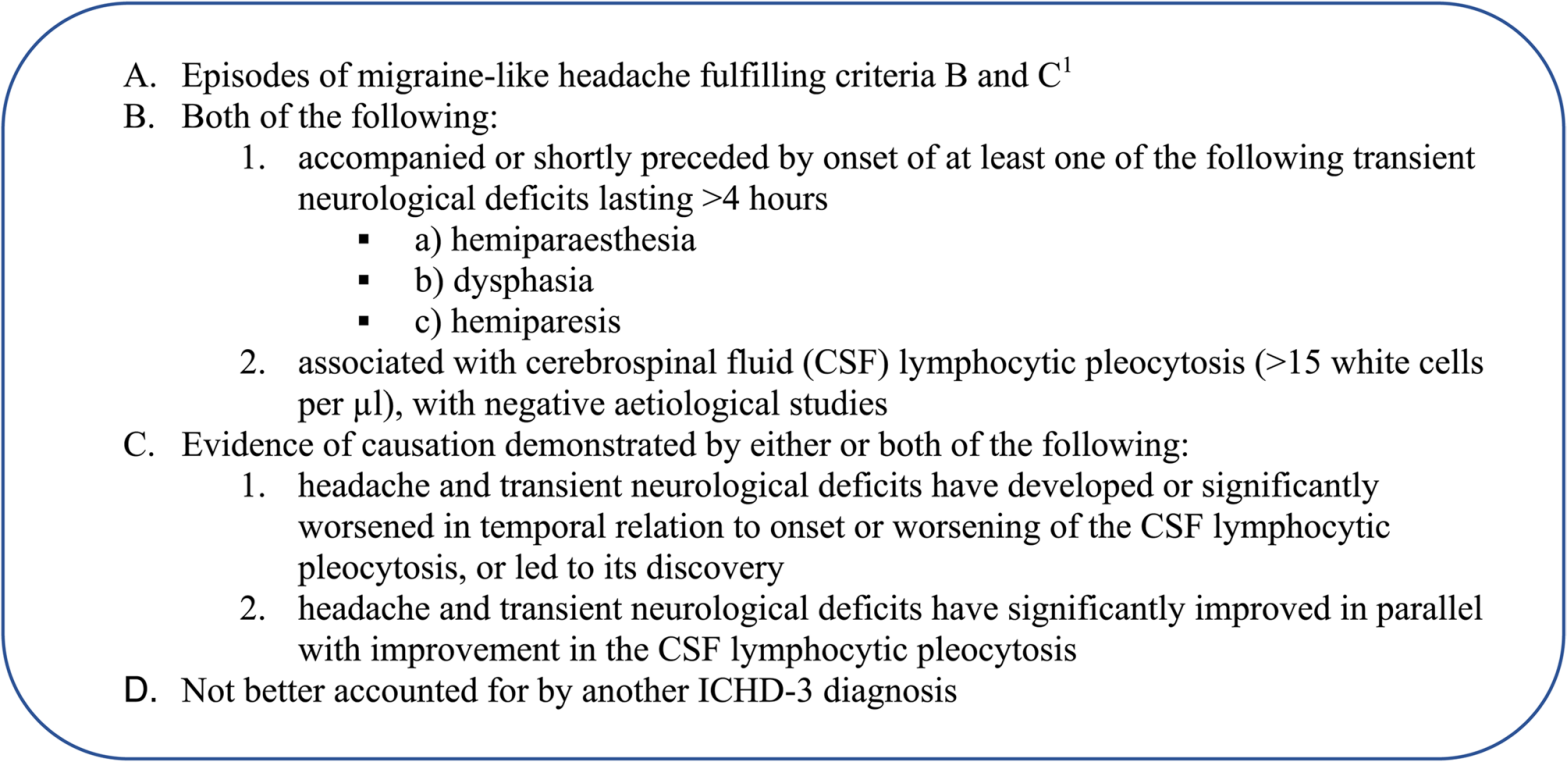
HaNDL diagnostic criteria according to ICHD-3 (cod. 7.3.5)

During hospitalization, the girl showed always good general conditions, reporting only a new episode of headache, without neurological symptoms, and fully responsive to paracetamol. Due to persistently negative neurological clinical examination, and negative imaging and laboratory tests, the diagnosis of HaNDL was made based on the International Classification of Headache Disorders 3rd edition (ICHD-3, Fig. 1) [1]. Since this form is associated with papilledema, the girl underwent ophthalmology evaluation, proving negative. She was discharged in good general condition, afebrile, and asymptomatic. She was recommended to keep a headache diary and to take prompt analgesic therapy on demand.

During the follow-up at our Pediatric Headache Center, the girl reported one single further episode of frontal, pulsating headache lasting about four hours, associated with photophobia, nausea and vomiting after paracetamol administration, followed by hypoesthesia of the left lower limb for about 15 min. The episode had spontaneous recovery.

## Narrative review

### Methods

To find literature on already described cases of HaNDL in children, we searched PubMed, Embase, Scopus and Google Scholar from inception to February 2025. We used the following terms combined with boolean operators OR: “HaNDL”; “Headache and Neurologic Deficits and cerebrospinal fluid Lymphocytosis”; “Headache with Neurologic Deficits and cerebrospinal fluid Lymphocytosis”; “migraine with cerebrospinal fluid pleocytosis”; “pseudomigraine with cerebrospinal fluid pleocytosis” (the latter being the original name used to describe HaNDL). On PubMed, we selected age limit “0–18 years”; on Embase we selected “adolescent”, “child”, “school”, and “preschool” limits; on Scopus and Google Scholar we added the terms “child*”, “pediatric*”, and “paediatric*” connected with boolean operators AND and OR.

**Fig. 2:**
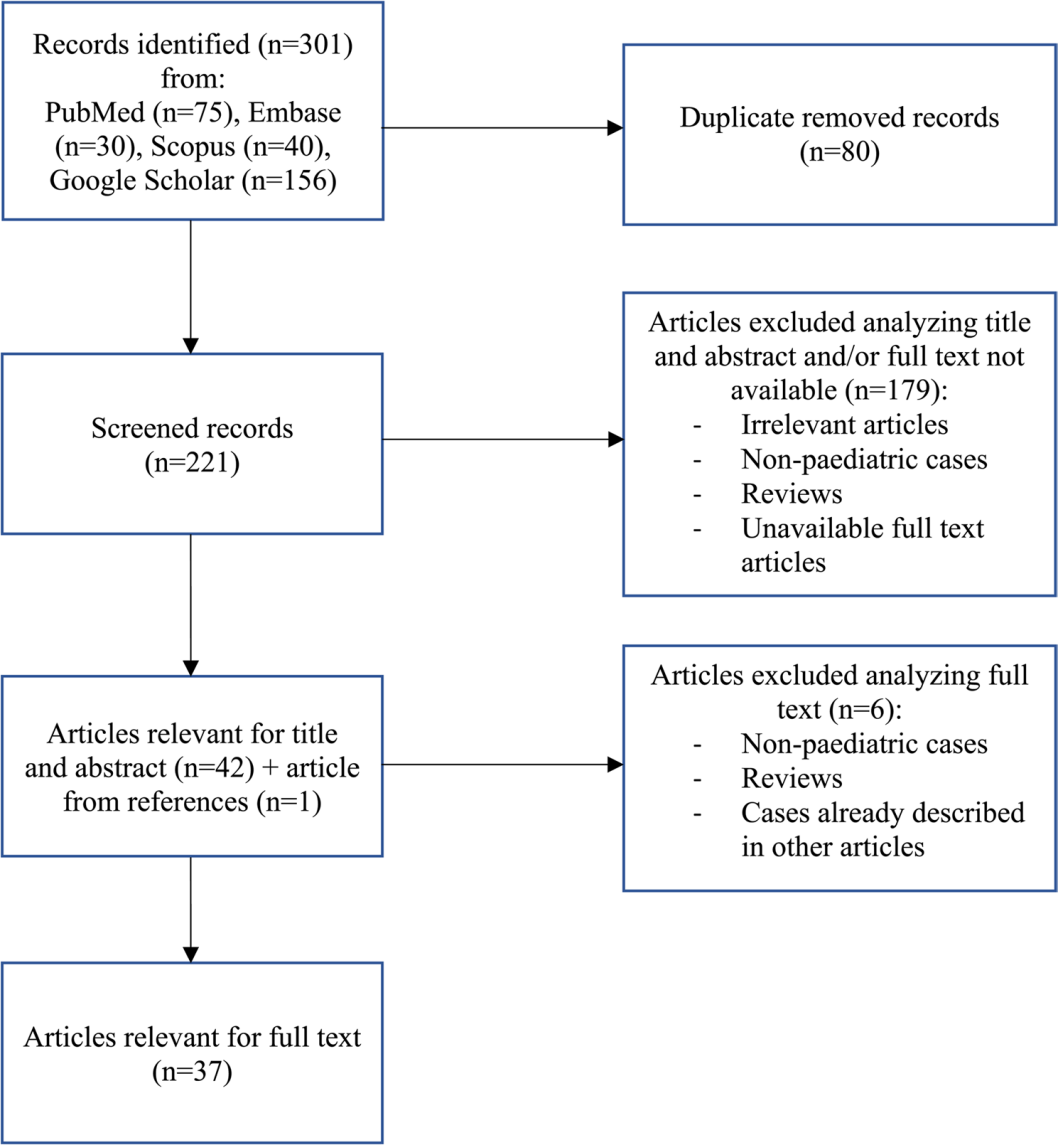
Flow-chart of literature review

We firstly found 301 eligible articles; after the selection process, 37 papers describing 43 children were finally included in our brief review (Fig. 2); our case was included, reaching 44 cases in total.

For each case included in this review, we collected data about age, sex, familiar and personal history of headache, past medical history, number of HaNDL episodes, duration, presence of viral prodrome (identified by viral symptoms with or without etiologic tests), characteristics of headache and neurologic symptoms, CSF analysis, diagnostic workout (EEG, CT, MRI, fundus oculi, infectious diseases and autoimmunity tests) and treatment. In case of more than one reported episode, we considered the longest duration of headache and neurologic symptoms; in case of more than one CSF analysis, we considered the highest value of pleocytosis; in case of more than one diagnostic test we considered the pathologic one.

The main features of all the patients included in this review are summarized in Table 1.

**Table 1:** Epidemiological, clinical and diagnostic features of all the 44 children with HaNDL syndrome included in the review

Patient	Age	Sex	HaNDL: Yes, Very probable, Quite probable	Sensory symptoms	Motor symptoms	Impaired speech	Other symptoms/ papilledema	Neurologic symptoms laterality	Headache quality/ Intensity if available	Headache laterality if available	WBC at CSF analysis/µl (% of lymphocytes) if available	Other alterations at CSF analysis	CSF opening pressure if increased (cm H2O)	EEG description if done	CT description if done	MRI description if done	Treatment	Outcome including number of episodes and duration of the syndrome if available
1 Armstrong-Javors [2]	16	M	Yes	Yes	Yes	Yes	Papilledema, Visual symptoms, Confusion, Impaired consciousness	R	Severe	L	303 (96%)	Proteinorrhachia (131 mg/dl)	35	Bilateral/ generalized slowing	-	Leptomeningeal enhancement	Emergency life support for elevated intracranial pressure, Symptomatic treatment	3 episodes in 6 days
2 Sultan [3]	14	M	Yes	Yes	Yes	Yes	Confusion	R	Throbbing	L	226 (100%)	Proteinorrhachia (90 mg/dl)	-	Unilateral slowing	Normal	Normal	-	2 episodes
3 Soto- Insuga [4]	14	F	Yes	Yes	Yes	Yes	Visual symptoms, Confusion, Fever	R	Throbbing, severe	Bilateral	250 (90%)	Proteinorrhachia (152 mg/dl)	-	Unilateral slowing	-	Normal	-	8 episodes in 2 months
4 Davidsen [5]	18	F	Very probable	Yes	Yes	No	Visual symptoms	L	Constricting, moderate	Bilateral	500	Proteinorrhachia (60 mg/dl)	-	Unilateral slowing	Normal	Normal	-	4 episodes in 3 weeks
5 Toth [6]	18	M	Yes	Yes	Yes	Yes	Visual symptoms, Confusion	Side alternance between episodes	Throbbing, severe	L	298 (97%)	Proteinorrhachia (125 mg/dl)	49.5	Bilateral/ generalized slowing	Normal	Normal	Symptomatic treatment	4 episodes in a month
6 Frediani [7]	18	M	Very probable	Yes	No	No	Confusion, Fever	Bilateral	-	-	22	Proteinorrhachia	-	Unilateral slowing	Normal	Normal	-	3 episodes in 3 years
7 Barroso [8]	17	F	Yes	Yes	Yes	Yes	Visual symptoms	R	Constricting, severe	L	252 (100%)	-	-	Bilateral/ generalized slowing	Normal	Normal	-	4 episodes in 10 days
8 Goncalves [9]	14	F	Very probable	Yes	No	Yes	Fever	L	Severe	Occipital	250 (90%)	-	-	-	Normal	Leptomeningeal enhancement attributed to prior lumbar puncture	-	2 episodes in 17 days
9 Garcia- Roves [10]	11	F	Yes	Yes	Yes	Yes	Confusion, Impaired consciousness, Fever	R	Throbbing, severe	-	187 (90%)	Proteinorrhachia (110 mg/dl)	-	Unilateral slowing	Normal	Normal	-	4 episodes in 10 days
10 Tsang [11]	18	F	Yes	Yes	No	Yes	-	L ->R	Severe	-	84 (100%)	Proteinorrhachia (74 mg/dl)	-	Bilateral/ generalized slowing	-	Normal	-	2 episodes in a month
11 Chan [12]	18	F	Yes	Yes	No	No	Papilledema, Visual symptoms, Fever	Bilateral	-	-	172 (100%)	Proteinorrhachia (92 mg/dl)	32	-	-	Normal	-	One episode, Prolonged visual impairment
12 Moavero [13]	17	F	Yes	No	No	Yes	Papilledema, Confusion, Impaired consciousness	Not applicable	Throbbing	-	246 (100%)	Proteinorrhachia (73 mg/dl), Increased liquor glucose (147 mg/dl)	65	-	-	Normal	-	One episode lasted 2 weeks
13 Moavero [13]	9	M	Very probable	No	No	No	Papilledema, Confusion	Not applicable	Throbbing	-	87 (100%)	Proteinorrhachia (79.9 mg/dl)	37	-	-	Normal	Symptomatic treatment	One episode lasted three weeks
14 Moavero [13]	14	M	Very probable	No	Yes	Yes	Papilledema	Side alternance in the same episode	Throbbing, severe	-	90 (90%)	-	33	-	-	Normal	Emergency life support for elevated intracranial pressure	2 episodes in 5 months
15 Mulroy [14]	16	F	Yes	Yes	No	Yes	Papilledema, Visual symptoms, Confusion	R	Severe	Generalized	110 (92%)	-	50	-	-	Normal	-	4 episodes in a month, Prolonged visual impairment
16 Filina [15]	16	M	Yes	No	Yes	Yes	Confusion	R	Severe	-	110 (98%)	Proteinorrhachia (83 mg/dl)	38	Unilateral slowing	Normal	Non-specific anomalies	-	2 episodes
17 Fumal [16]	16	F	Very probable	Yes	Yes	No	Visual symptoms	L	Throbbing	R	260 (95%)	Proteinorrhachia (90 mg/dl)	-	Unilateral slowing	Normal	Normal	-	5 episodes
18 Guillan [17]	16	F	Yes	No	No	Yes	-	Not applicable	Throbbing, severe	-	lymphocytic pleocytosis	Proteinorrhachia	-	Performed, result not available	Normal	Normal	-	2 episodes in 3 days
19 Guillan [17]	15	M	Very probable	No	Yes	Yes	-	R	Throbbing, severe	-	Not available	Proteinorrhachia	-	Performed, result not available	Normal	Normal	-	9 episodes
20 Berthold [18]	12	M	Yes	No	No	Yes	Visual symptoms, Fever	Not applicable	Severe	Bilateral	69 (100%)	Proteinorrhachia (53.9 mg/dl)	-	--	-	Normal	-	3 episodes in a month
21 Armiento [19]	12	F	Yes	No	Yes	Yes	Visual symptoms, Impaired consciousness	Bilateral	Throbbing, severe	-	48 (94%)	Increased liquor glucose (76 mg/dl)	-	-	Normal	Normal	Emergency life support for elevated intracranial pressure	One episode lasted 3 days
22 Morrison [20]	17	F	Yes	Yes	No	Yes	Papilledema, Visual symptoms	L	Throbbing	Bilateral	169 (100%)	Proteinorrhachia (97 mg/dl)	47	-	-	Normal	Symptomatic treatment	One episode lasted 2 weeks
23 Morrison [20]	16	F	Very probable	No	Yes	No	Papilledema, Visual symptoms	L	-	-	12	-	55	-	Normal	-	-	One episode lasted 35 days, Prolonged visual impairment
24 Emond [21]	18	M	Quite probable	Yes	Yes	Yes	-	R	Throbbing, severe	-	400 (100%)	Proteinorrhachia (200 mg/dl)	-	Unilateral slowing	Normal	Leptomeningeal enhancement	-	9 episodes in 20 days
25 Rivero- Sanz [22]	14	F	Very probable	Yes	Yes	Yes	Papilledema, Visual symptoms	R	Severe	Occipital	490 (90%)	-	31	Unilateral slowing	-	Normal	Symptomatic treatment	Not available
26 Bartleson [23]	16	F	Yes	Yes	Yes	Yes	Visual symptoms, Confusion, Impaired consciousness	Side alternance between episodes	Throbbing, severe	L	156 (99%)	Proteinorrhachia (100 mg/dl), Increased liquor glucose (93 mg/dl)	-	Unilateral slowing	Normal	-	-	3 episodes in a week
27 Bartleson [23]	17	M	Very probable	Not available	Not available	Not available	-	Not available	-	-	37	Proteinorrhachia (87 mg/dl)	-	Bilateral/ generalized slowing	Normal	-	-	6 episodes in a week
28 Bartleson [23]	17	F	Very probable	Not available	Not available	Not available	Fever	Not available	-	-	73	Proteinorrhachia (90 mg/dl)	29	Bilateral/ generalized slowing	Normal	-	-	8 episodes in 3 weeks
29 Safier [24]	10	F	Yes	Yes	No	Yes	Confusion, Impaired consciousness	L	Severe	-	372 (92%)	Proteinorrhachia (151 mg/dl)	-	Bilateral/ generalized slowing	-	Normal	Emergency life support for elevated intracranial pressure, Symptomatic treatment	5 episodes in a month
30 Fernandez-Rodriguez [25]	14	F	Yes	Yes	No	Yes	Confusion	R	Throbbing	-	148 (99%)	-	-	Unilateral slowing	Normal	Normal	-	3 episodes in a week
31 Garcia [26]	14	M	Yes	Yes	No	Yes	Visual symptoms	L	Throbbing, moderate	Generalized	525 (99%)	Proteinorrhachia (98.3 mg/dl)		Normal	Normal	Normal	-	3 episodes
32 Anger [27]	10	F	Yes	No	Yes	No	Papilledema, Visual symptoms	R	-	-	lymphocytic pleocytosis	-	-	Unilateral slowing	-	Non-specific anomalies	-	2 episodes in 2 weeks
33 Taha [28]	13	M	Very probable	Yes	Yes	Yes	Visual symptoms, Confusion	Not available	Throbbing, severe	-	195 (lymphocyte predominance)	-	31	Unilateral slowing	-	Normal	-	Not available
34 Saini [29]	5	M	Yes	Yes	No	No	-	Bilateral	Continuous, severe	Generalized	60 (100%)	-	-	-	-	Normal	-	3 episodes in 6 months
35 Dhawan [30]	11	M	Very probable	Yes	No	No	Visual symptoms	Bilateral	Continuous, severe	-	200 (lymphocyte predominance)	Proteinorrhachia (45 mg/dl)	-	-	-	Normal	Symptomatic treatment	3 episodes in 2 years
36 Suresh [31]	8	F	Very probable	No	Yes	No	Visual symptoms, Seizures, Fever	Bilateral	Throbbing, severe	Generalized	18 (100%)	Proteinorrhachia (71 mg/dl)	-	Bilateral/ generalized slowing	-	Normal	Symptomatic treatment	6 episodes in 10 days
37 Al Towairqui [32]	18	M	Yes	Yes	No	No	Visual symptoms	Side alternance between episodes	Throbbing	-	100 (98%)	Proteinorrhachia (50 mg/dl)	-	Unilateral slowing	-	Non-specific anomalies	Symptomatic treatment	2 episodes in 2 weeks
38 Rathore [33]	13	F	Quite probable	No	No	No	Seizures, Fever	Not applicable	Severe	-	34 (lymphocyte predominance)	Proteinorrhachia (103 mg/dl)	-	Unilateral slowing	Normal	Leptomeningeal enhancement	Symptomatic treatment	2 episodes
39 Vermaning [34]	10	F	Very probable	Yes	Yes	Yes	Papilledema, Impaired consciousness	L	-	R	210 (94.3%)	-	-	Unilateral slowing	Normal	Non-specific anomalies	Symptomatic treatment	3 episodes in 35 days
40 Samanta [35]	16	F	Very probable	Yes	No	No	Visual symptoms	L ->R	Stabbing, severe	Generalized	lymphocytic pleocytosis	-	38	Bilateral/ generalized slowing	-	Normal	-	One episode lasted 10 days
41 Gabaldon- Albero [36]	12	F	Yes	Yes	Yes	Yes	Visual symptoms, Impaired consciousness	R ->L	Severe	Bilateral	479 (100%)	Proteinorrhachia (175.8 mg/dl)	40	Normal	Normal	Normal	Symptomatic treatment	2 episodes in 12 days
42 Dores [37]	17	M	Yes	Yes	No	Yes	Visual symptoms, Confusion	R	Constricting, severe	L	128 (lympho-monocytosis)	Proteinorrhachia (70.7 mg/dl)	-	Unilateral slowing	Normal	Normal	Symptomatic treatment	3 episodes in 49 days
43 La Malfa [38]	11	M	Yes	Yes	Yes	Yes	Confusion	R	-	-	591 (99%)	Proteinorrhachia (75 mg/dl)	-	Unilateral slowing	Normal	Leptomeningeal enhancement	-	One episode lasted a day
44 Our case	15	F	Very probable	Yes	No	Yes	-	R	Throbbing, moderate	L	127 (100%)	Proteinorrhachia (62.2 mg/dl)	-	Normal	Normal	Normal	Symptomatic treatment	2 episodes in 2 months

*M* male, *F* female, *R* right, *L* left, *L* ->*R* left symptoms spreading right secondary, *R* ->*L* right symptoms spreading left secondary, *WBC* white blood cells, *CSF* cerebrospinal fluid, *EEG* electroencephalogram, *CT* computed tomography, *MRI* magnetic resonance imaging

### Epidemiology and clinical presentation

Among the 44 paediatric cases of HaNDL described so far to our knowledge, 25 fulfilled all HaNDL diagnostic criteria according to ICHD-3 [2–4, 6, 14, 15, 27, 29, 37]. Seventeen other children (including ours) were very probable cases of HaNDL, as they fulfilled all but one criterion (in detail: shorter duration of neurological symptoms [5, 9, 16, 17, 22, 28], symptoms duration not reported [13, 23, 30, 34, 35], different neurologic symptoms from the classic triad [13], lack of temporal link between symptoms and lumbar puncture [7], presence of less than 15 cells/µl at CSF analysis (12 cell/µl) [20], positive Dengue sierology [31]). Finally, 2 children were quite probable cases of HaNDL: they both had shorter duration of symptoms, one of them had positive HHV6 serology [21], and the other one had different neurologic symptoms from the classic triad [33].

As for other types of headaches, ICHD-3 diagnostic criteria for HaNDL syndrome are often not completely fulfilled in children at the time of first clinical presentation, but could develop over time, thus adequate follow-up is crucial to achieve the diagnosis. Moreover, ICHD-3 classification was primary created for adult patients and reports pediatric-specific features only for some diagnostic categories. In particular, ICHD-3 does not report any specific pediatric characteristic about HaNDL syndrome. Such issue underlines the strict necessity of collecting large case series of affected children to better define age-specific features. A previous study estimated that among about 200 cases of HaNDL described in literature, only 15% involved children [2]. In our review exclusively on pediatric cases, children’s median age was 15 years (range 5–18 years). We found female predominance (26/44, 59.1%), in contrast with previous literature mainly on adult cases, where consistent sex predominance was not reported [39, 40]. Twelve patients (27.3%) had familiar history for headache, and 8 patients (18.2%) reported personal history of headache, in agreement with data reported in the ICDH-3 [1]. Only one girl had remarkable personal history (type 1 diabetes mellitus), while diabetes mellitus and hypertensions were described in a small number of patients in a previous review including both adults and children [41]. 

Among the 42 children with exactly known clinical features, the most frequent neurological symptoms were sensory (paresthesia, dysesthesia; 71.4%), followed by impaired speech (dysphasia, aphasia, dysarthria; 69.0%) and motor symptoms (paresis, weakness; 52.4%). In 15 cases neurological signs and symptoms had right laterality, and in 8 cases left laterality. Ten patients presented with bilateral signs and symptoms (Table 1) and in 3 cases there was side alternance between the episodes. In the remaining cases, laterality was unknown or not applicable. Predominance of sensory symptoms (followed by aphasic and motor symptoms) and right laterality has been described in adults as well [1, 40, 42]. 

As already described in literature, HaNDL can also present with visual symptoms [40]: they were found in 23 patients of our review, and they varied from decreased vision, visual field loss, ophthalmoplegia, diplopia and photophobia. Despite the focal character of the syndrome, we found more diffuse manifestations as well. Confusional state, already described in adults [43, 44], has been found in 16 children; fever, impaired consciousness and seizures were reported respectively in 9, 8 and 2 cases. Besides these children, one additional case of HaNDL with seizures has been reported in a 35-year-old male [45]. 

HaNDL can be also associated with papilledema [1, 41]: eleven children (25.0%) presented this condition; this is a remarkable number, especially considering that only 17 patients underwent ophthalmological evaluation.

According to literature, headache of HaNDL is moderate to severe, and usually throbbing in quality [41]. In our review, headache quality was described in 26 patients, and defined throbbing by 20 of them (76.9%). Other less mentioned features were constricting, continuous, and stabbing headache. Headache intensity was reported in 29 cases, being severe in 89.7% and moderate in 10.3%. When described, headache quality, intensity and laterality are reported in Table 1.

Duration of headache and of neurologic symptoms was difficult to resume because of data heterogeneity: they could last from minutes to weeks. According to ICHD-3, most of the episodes lasts for 2 h, possibly lasting more than 24 h [1]. In our study, the mean number of episodes was 3.2 per patient and the syndrome had a mean duration of 2.7 months per patient. Even if HaNDL is considered a monophasic disorder, most of patients present with more than one attack [39]. The syndrome resolves usually within three months [1]. 

### Etiology and pathogenesis

The etiology of HaNDL is currently unknown, but autoimmune mechanisms have been proposed and investigated in some cases reported in literature. It has been supposed that a viral infection leads to production of antibodies that react against neuronal or vascular antigens, leading to an aseptic leptomeningeal vasculitis and subsequently headache and neurologic symptoms via a spreading depression-like mechanism [46]. In a study comparing 5 HaNDL adult patients to 30 controls, microarray and immunoprecipitation methods detected antibodies against three DNA repair proteins (mitogen-activated protein kinase-4, DNA-dependent protein kinase catalytic subunit, DNA excision repair protein ERCC-6) in 3 sera from HaNDL patients and in none of controls [47]. Other studies detected antibodies against P/Q type voltage-gated calcium channel (VGCC), CACNA1A [48] and CACNA1H [49]. In particular, CACNA1A mutation is a diagnostic criterion for Familial Hemiplegic migraine type 1, however, this mutation has been excluded in many patients with HaNDL [1]. Inflammatory or infectious origin is supported by CSF lymphocytosis, the monophasic course of disease, and viral prodrome [23, 39]. In our review, 9 patients had a viral prodrome (20.5%): in particular, some authors documented positive serologies for recent HHV6 infection [21], Dengue [31], and COVID-19 [26]. Our girl reported history of recent gastroenteritis, but infectious disease workout proved negative.

Transcranial doppler sonography performed in two patients during and after HaNDL attacks showed asymmetrical fluctuations in middle cerebral arterial blood flow velocity and pulsatility, indicating that intracranial vasomotor changes play a role in the pathophysiology of HaNDL [50]. Single photon emission computed tomography (SPECT) studies on HaNDL patients showed a reduction in brain blood flow on the side of origin of neurological signs and symptoms [10, 25, 51]; hemispheric hypoperfusion was documented at CT [52] and MRI [53]. These findings suggest a spreading depression-like mechanism, recalling migraine pathophysiology [51, 52]. The latter is supported also by electrophysiological findings (single-fiber electromyography and visual evoked potentials) in a 16 year-old boy, which presented similar characteristics of patients suffering from migraine with aura [24]. 

### Diagnosis and differential diagnosis

HaNDL is a diagnosis of exclusion. Differential diagnosis should consider stroke, tumors, epilepsia, neuro-infective disorders, autoimmune encephalitis, vasculitis, hemiplegic migraine and migraine with aura [2, 36]. Given the usual onset of HaNDL with severe headache and neurologic symptoms, an extensive work-up including CSF analysis, neuroimaging and EEG is necessary in order to exclude serious and urgent conditions [46]. Focal neurologic signs and symptoms can mimic stroke and transitory ischemic attacks, even if HaNDL is more likely to present with a slow evolution over several minutes rather than with abrupt onset.

Cerebrospinal fluid lymphocytic pleocytosis is a diagnostic criterion of the syndrome [1]: in our review the mean value of white blood cells at CSF analysis was 201.1/µl, with mean percentage of lymphocytes of 97%. Proteinorrhachia is a common finding in HaNDL [40, 41], as well as elevated CSF opening pressure [40]. In our population 31 patients (70.5%) had increased liquor proteins (mean value 96.1 mg/dl). Increased liquor glucose has been found in 3 cases (mean value 105,3 mg/dl). Fifteen children showed also increased opening pressure at lumbar puncture (34.1%), and 40.7 cmH_2_O was the mean pressure among altered cases. However, data about CSF opening pressure in childhood are scanty [54, 55], and independent factors may heighten its value, such as sedation-related hypercapnia and crying in not sedated children [55]. In our case, CSF opening pressure was not measured.

EEG was asked for 32 children and showed anomalies in 27 cases (84.4%). According to previous studies [39–41], the most common finding was slow activity with delta waves: unilateral slowing (18/27) was more common than bilateral (9/27). Anyway, EEG pathologic findings did not always correlate to the topography of focal symptoms [56]. EEG can also help excluding epilepsia, though HaNDL episodes are often too long to be epileptic [2]. 

Neuroimaging is crucial: in case of stroke both perfusion and diffusion are impaired, while in HaNDL there is a perfusion/diffusion mismatch. The mismatch pattern, without perfusion changes corresponding to a vascular territory is suggestive of oligoemia rather than ischemia [2, 52, 57]. Although angiography may be necessary to exclude arteriopathies, some authors recommend cautions, because in HaNDL patients angiography may precipitate symptoms and predispose cerebral infarction, especially because the risks of angiography increase after a migraine attack [23, 57]. In HaNDL, CT, MRI (with or without contrast) and angiography are usually normal in the inter-critic period, but during an episode they can show delayed brain perfusion without increased diffusion-weighted imaging changes, and narrowing of cerebral arteries [1]. Non-specific findings such as white matter lesions, meningeal enhancement [41], and small areas of high signal [46] can be found as well. In our review, 25 children underwent CT, all proving negative, and 40 underwent MRI, showing non-specific anomalies in 9 cases. In particular, 5 children had leptomeningeal enhancement, which was attributed to prior lumbar puncture in one case (Table 1) [9]. 

Unlike children suffering from infectious encephalitis, patients with HaNDL are asymptomatic with a normal mental status between episodes, they do not show meningeal signs, and do not require treatment for symptom resolution [2]. On the other hand, mild or incomplete forms of anti-NMDA receptor encephalitis mimicking HaNDL have been described, though it usually shows as severe multistage neuropsychiatric syndrome. For this reason, NMDAR antibodies detection is important to distinguish between the two syndromes, as HaNDL does not appear to be mediated by anti-NMDAR antibodies [58]. In our review the majority of patients (41/44) underwent cerebrospinal fluid and/or blood tests to rule out infectious diseases: pathogens were always negative on cerebrospinal fluid; three children had serologies suggestive for recent viral infection (Dengue and HHV6, respectively) [21, 31], and one patient had documented history of SARS-CoV-2 infection in the previous month [26]. Autoimmunity tests on serum and/or cerebrospinal fluid were done on 30 patients, with negatives results in all cases but one with positive calcium channel binding antibody, N-type [27]. 

HaNDL may also mimic primary CNS vasculitis, as both are characterized by focal neurologic signs and symptoms, with similar cerebrospinal fluid profile. At neuroimaging, leptomeningeal enhancement can occur in both conditions, but parenchymal changes due to multifocal inflammatory lesions are more common in CNS vasculitis [2]. 

Recurrent pleocytosis observed in HaNDL patients is reported also in Mollaret meningitis, a form of recurrent benign lymphocytic meningitis which presents with episodic mild fever, sign and symptoms of meningeal irritation, photophobia, headache, and impairment in mental status. Characteristic CSF findings include marked pleocytosis with neutrophils, lymphocytes, and large mononuclear (Mollaret) cells, that are typical for this disease, but not for HaNDL [9, 15]. 

Finally, HaNDL might be misdiagnosed with migraine with aura or without aura and with hemiplegic migraine: however, CSF pleocytosis does not occur in these conditions [39, 53]. 

### Treatment and outcome

HaNDL is a self-limiting syndrome, usually showing spontaneous resolution within 3 months [1]. During this period, the cornerstones of management are symptomatic treatment, reassurance of both patients and their families, and prompt interception of any episode following the first one.

In our review, symptomatic treatment was reported only for 14 patients (31.8%), and 32 children have been followed after the first episode (72.7%). Overall, NSAIDs were the most prescribed analgesics (78.6%), followed by paracetamol (42.9%); NSAIDs and/or paracetamol were reported to be effective in 9 cases. Triptans and metamizole were prescribed in two cases each. Prophylaxis was prescribed with flunarizine in three cases and with amitriptyline in one case. Acetazolamide was prescribed in 6 cases and was reported to be effective in 4/6 cases.

Probably, the rate of children under symptomatic treatment is underestimated. On the other hand, many children have been treated with antibiotics, antiviral and steroidal drugs before reaching the correct diagnosis of HaNDL: this observation suggests that better knowledge of the syndrome may help clinicians not to use inappropriate therapies. In particular, information about antimicrobial and antiviral therapy was reported for 23 patients (52.3%): ceftriaxone was the most prescribed antibiotic (34.8%), while cefixime, amikacin and vancomycin were reported in one case each; acyclovir the most used antiviral agent (78.3%). In all the other cases, antimicrobial therapy was not specified. The duration of the treatment was reported only in 6 cases, ranging from 12 h to 14 days, anyway in most cases antimicrobial and antiviral therapy was suspended when infections were ruled out.

Unfortunately, available data about empirical antimicrobial and antiviral therapy from our review are insufficient to draw a comprehensive picture. Anyway, HaNDL is an exclusion diagnosis, thus empirical therapy should not be responsible of delayed diagnosis. On the other hand, pediatricians should include HaNDL syndrome among possible differential diagnoses in children with suggestive clinical picture.

Available literature about clinical features of children on follow-up and even about follow-up duration are scanty. Though the prognosis is generally favorable, clinicians should be aware that a subgroup of patients do not follow the generally benign course of the syndrome and may suffer from complications. In particular, some patients could experience prolonged visual impairment due to elevated intracranial pressure [2], which has been reported by three children in our review [12, 14, 20]. Moreover, elevated intracranial pressure could require emergency life support (as reported both for some adults [59] and children, [2, 13, 19, 24]), CSF drainage, and therapy with acetazolamide [14, 59]. 

Due to the lack of specific recommendations, strict follow-up should be done in the first 3 months since the onset and could be prolonged until the first year. After that, the follow-up could be tailored case by case. In case of recurrent attacks within 3 months since the onset, some authors suggest repeating head CT and MRI for each attack and CSF analysis only for the second one, if the syndrome is well-established; more extensive diagnostic tests may be necessary if the diagnosis is not well-established [46]. 

## Conclusions

HaNDL is a rare syndrome affecting both adults and children. Our paper describes a pediatric case of HaNDL and summarizes the characteristics of the syndrome in children through the review of the 44 cases reported in literature so far.

While no sex predominance is reported in adults, females prevail in childhood, and most cases are reported in adolescents. HaNDL presentation includes headache and neurologic signs and symptoms (hemiparesthesia, dysphasia and hemiplegia). As for adults, the most frequent neurological symptoms in children are sensory, while confusional state is reported only in one third of pediatric patients. The etiology is currently unknown, but autoimmune mechanisms have been proposed. The diagnostic work out includes CSF analysis (with detection of lymphocytosis and possibly proteinorrachia and increased CSF pressure), neuroimaging (proving negative in the intercritical period, possibly with delayed cerebral perfusion and narrowing of cerebral arteries during an episode) and EEG (possibly showing focal findings in the symptomatic period). Proteinorrhachia is a common finding in pediatric HaNDL as well as elevated CSF opening pressure; however, data about CSF opening pressure in childhood are scanty. Differential diagnosis should consider stroke, tumors, seizures, neuro-infective disorders, autoimmune encephalitis, vasculitis, hemiplegic migraine and migraine with aura. Though the treatment is symptomatic, many children are treated with antibiotics, antiviral and cortisonic drugs before reaching the correct diagnosis of HaNDL syndrome. As for adults, elevated intracranial pressure could require emergency life support, CSF drainage, and/or acetazolamide. The course of the syndrome is self-limiting, usually with resolution within 3 months. Even if HaNDL is considered a monophasic disorder, most of children present with more than one attack, thus careful follow-up is crucial. Pediatric headache specialists are aware of the peculiarity of clinical presentation of the different types of headaches (including HaNDL) in childhood compared to adults. For this reason, children with HaNDL syndrome should be referred to dedicated pediatric headache centres.

## Supplementary Information

Supplementary Material 1.

## Data Availability

Not applicable.

## Abbreviations

HaNDL: Headache and Neurological Deficit with cerebrospinal fluid Lymphocytosis
ED: Emergency department
CT: Computed tomography
EEG: Electroencephalogram
MRI: Magnetic resonance imaging
ICHD-3: International classification of headache disorders 3rd edition
CSF: Cerebrospinal fluid
SPECT: Single photon emission computed tomography
