# Supplementary material for: Transient Headache and Neurological Deficits with cerebrospinal fluid Lymphocytosis (HaNDL) syndrome in children: case report and narrative review
Source: Ital J Pediatr. 2025 Nov 19;51:306. doi: 10.1186/s13052-025-02139-9 (PMC12629036; doi:10.1186/s13052-025-02139-9)
Supplement: Supplementary file 1 — Supplementary Material 1. [file 13052_2025_2139_MOESM1_ESM.doc]

# **Manuscript ITJP-D-25-00385R1**

Dear Editor,

Thank you for your letter. We have revised the article following the reviewers’ suggestion.

In particular:

**Reviewer 1**

**The authors adequately addressed all comments. This revised manusxript now is recommended for acceptance.**

Thank you for your appreciation.

**Reviewer 2**

**Overall, the article has been comprehensively revised under the reviewer's suggestions, enhancing the readability of the data and the clarity of the arguments. Meanwhile, the accuracy of terminology and content has been improved. It can be considered that the revised version is relatively complete. It is recommended to improve Table 1, and use "-" to represent the blank spaces.**

Thank you for your comment. As recommended, we have filled blank cells of Table 1 with “-“.
